# Paradoxical effect of IKKβ inhibition on the expression of E3 ubiquitin ligases and unloading‐induced skeletal muscle atrophy

**DOI:** 10.14814/phy2.13291

**Published:** 2017-08-24

**Authors:** Svetlana P. Belova, Boris S. Shenkman, Tatiana Y. Kostrominova, Tatiana L. Nemirovskaya

**Affiliations:** ^1^ Institute of Biomedical Problems, RAS Moscow Russia; ^2^ Department of Anatomy and Cell Biology Indiana University School of Medicine‐Northwest Gary Indiana; ^3^ Faculty of Basic Medicine Lomonosov Moscow State University Moscow Russia

**Keywords:** FoxO3, MAFbx, MuRF1, nuclear factor‐*κ*B, skeletal muscle, unloading

## Abstract

We tested whether NF‐*κ*B pathway is indispensable for the increase in expression of E3‐ligases and unloading‐induced muscle atrophy using IKKβ inhibitor IMD‐0354. Three groups of rats were used: nontreated control (C), 3 days of unloading/hindlimb suspension with (HS+IMD) or without (HS) IMD‐0354. Levels of I*κ*B*α* were higher in HS+IMD (1.16‐fold) and lower in HS (0.82‐fold) when compared with C group. IMD‐0354 treatment during unloading: had no effect on loss of muscle mass; increased mRNA levels of MuRF1 and MAFbx; increased levels of pFoxO3; and had no effect on levels of Bcl‐3, p105, and p50 proteins. Our study for the first time showed that inhibiting IKK *β* in vivo during 3‐day unloading failed to diminish expression of ubiquitin ligases and prevent muscle atrophy.

## Introduction

Activation of nuclear factor‐*κ*B (NF‐*κ*B) pathway is important for skeletal muscle atrophy in response to diverse physiological and pathological stimuli including muscle unloading (Jackman et al. 2013; Bodine and Baehr 2014). Skeletal muscle‐specific E3 ubiquitin ligases MuRF1 and MAFbx are upregulated during unloading‐induced muscle atrophy. Both NF‐*κ*B and transcription factor FoxO3 can activate MuRF1 and MAFbx expression in response to unloading and immobilization. Moreover, NF‐*κ*B and FoxO3 have an additive effect on atrophy (Reed et al. 2011), suggesting that they both are important for activation of ubiquitin ligases (Bodine and Baehr 2014). Dephosphorylation of FoxO3 promotes its translocation into the nuclei and activation of MuRF1 expression (Lokireddy et al. 2012). Nevertheless, a recent study by Wu et al. (2014) reports that NF‐*κ*B sites, but not FoxO sites, are required for MuRF1 promoter activation in rat soleus muscles after 5 days of hindlimb unloading. The review by Jackman et al. (2013) suggests that various factors of the NF‐*κ*B signaling pathway are activated during muscle atrophy caused by numerous diseases and by unloading‐induced muscle atrophy.

Expression of dominant‐negative IkB kinase alpha (IKK*α*) or I*κ*B kinase beta (IKK*β*) inhibits NF‐*κ*B during unloading and decreases skeletal muscle fiber atrophy by 50% (van Gammeren et al. 2009). Expression of both IKK*α* and IKK*β* has an additive effect and decreases skeletal muscle fiber atrophy by 70% (van Gammeren et al. 2009). In mice with muscle‐specific IKK*β* deficiency denervation‐induced skeletal muscle atrophy was decreased (Mourkioti et al. 2006). Unloading‐induced activation of the MuRF1 expression requires putative NF‐*κ*B sites in the MuRF1 promoter region (Wu et al. 2014). To the contrary, removal of all three of the FoxO putative sites in the MuRF1 promoter region had no effect on the expression (Wu et al. 2014). These data indicate that IKK*β* and IKK*α* are required for the activation of MuRF1 and unloading‐induced skeletal muscle atrophy.

In the previous experiments the role of NF‐*κ*B pathway during unloading‐induced muscle atrophy was tested using knockout mice or by analyzing muscles after transfection with plasmids (Mourkioti et al. 2006; van Gammeren et al. 2009; Jackman et al. 2013). It is known that knockout mice can develop adaptations compensating for the lack of particular gene and often are not presented with pathological changes that are found in humans when the same gene is missing or mutated (Xu et al. 2015).

In difference from previous investigations, in this study we tested whether a pharmacological inhibition of IKK*β* in vivo can prevent unloading‐induced skeletal muscle atrophy and whether this involves decreased activation of MuRF1 and MAFbx transcription factors. We used IMD‐0354, a selective inhibitor of IKK*β* that suppresses NF‐*κ*B pathway‐dependent transcriptional activity (McFarlane et al. 2006). Mechanistically, IMD‐0354 blocks phosphorylation of IKK*β*, diminishes degradation of nuclear factor of kappa light polypeptide gene enhancer in B‐cells inhibitor alpha (I*κ*B*α*), and prevents translocation of NF‐*κ*B transcription factors into the nuclei and activation of MuRF1expression (Rothwarf and Karin 1999; Kaisari et al. 2013). We selected 3 days of unloading for our experimental conditions because the highest activation of MuRF1expression in skeletal muscle was observed at this time (Smith et al. 2014) and IκB*α* level was decreased (Judge et al. 2007). In this study, we for the first time tested the ability of IMD‐0354 to block I*κ*B*α* phosphorylation/degradation in vivo during muscle unloading. This approach allows better elucidation of the mechanisms regulating NF‐*κ*B signaling pathway as an important clinical target for the future treatment of skeletal muscle atrophy. We hypothesized that as NF‐*κ*B signaling plays a key role in the upregulation of E3 ubiquitin ligases during unloading therefore blocking IKK*β* will diminish muscle atrophy. Paradoxically our study showed that inhibition of IKK*β* in vivo using IMD‐0354 failed to diminish expression of MuRF1 and MAFbx ubiquitin ligases and unloading‐induced atrophy of soleus muscle.

## Materials and Methods

### Animal procedures

The experiments were performed in accordance with internationally accepted regulations and rules of biomedical ethics and were approved by the Committee on Bioethics of the Russian Academy of Sciences (protocol 314, 03.06.2013). All animals were kept at 22°C in a light‐controlled environment (12:12 h light‐dark cycle) with water and food available ad libitum. Twenty‐one male Wistar rats (3 months old, 200–239 g body weight range) were randomly assigned to one of the three groups with seven animals/group: nontreated control (C), 3 days of hindlimb suspension/unloading with (HS+IMD) or without (HS) supplementation with IKK*β* inhibitor IMD‐0354 (Sigma, St Louis, MO, USA). IMD‐0354 is a selective IKK*β* inhibitor that blocks I*κ*B*α* phosphorylation (IC_50_ ~ 250 nmol/L). IMD‐0354 was dissolved in 1% DMSO/saline and administered intramuscular into soleus muscle in concentration of 10 mg/kg of body weight per day using Hamilton syringe with a very fine needle. The IMD‐0354 dose used in our experiments is comparable to previously published data (Hosokawa et al. 2013). The control animals received equal volumes of 1% DMSO/saline vehicle solution using Hamilton syringe with a very fine needle. The first IMD‐0354 (HS+IMD group) or DMSO (C and HS groups) injection was performed 4 h prior to the hindlimb suspension. Subsequently, all rats were injected twice per day (morning and evening) during the entire duration of the experiments. Similar injection protocol was used in our previous publication (Shenkman et al. 2015). Visual inspection of the dissected soleus muscle showed no obvious signs of damage from the injection. Soleus muscles from the control rats that were subjected to the analogous injection protocol as the experimental animals showed no activation of E3 ubiquitin ligases or NF‐kB signaling. This suggests that minimal muscle damage that might have occurred due to the injection procedure had no influence on the results of this study. At the end of the experiment, rats were euthanized by overdose of sodium pentobarbital and soleus muscle was immediately dissected, weighed, divided into aliquots, frozen in isopentane cooled by liquid nitrogen, and stored at −85°C for the subsequent analyses.

### Hindlimb suspension protocol

The tail‐traction method of noninvasive tail‐casting procedure was used for the hindlimb suspension as previously described (Lomonosova et al. 2012). This technique used a swivel harness system incorporated into the casting materials, which was attached to a hook at the top of the cage. The hook was adjusted to allow only the forelimbs of the animal to reach the floor of the cage with the hindlimbs suspended; the body axis was at a 45 angle to the cage floor. Suspended animals were free to move around the cage using their forelimbs to obtain food and water.

### Protein extraction and Western blot analysis

Frozen soleus muscle samples were homogenized in ice‐cold RIPA lysis buffer (#SC‐24948, Santa Cruz) containing 50 mmol/L Tris (pH 7.4), 150 mmol/L NaCl, 0.1% Triton X‐100, 0.1% SDS, 5 mmol/L EDTA (pH 8.0), 1 mmol/L DTT, 1 mmol/L PMSF, 1 mmol/L Na3VO4, 10 *μ*g/mL aprotinin, 10 *μ*g/mL leupeptin, 10 *μ*g/mL pepstatin A, phosphatase inhibitor cocktail (#sc‐45045, Santa‐Cruz), and complete protease inhibitor cocktail (#sc‐29130, Santa‐Cruz, USA) as previously described (Lomonosova et al. 2012). Cytoplasmic and nuclear extracts were prepared from 50 mg of frozen soleus muscle using NE‐PER Nuclear and Cytoplasmic Extraction Reagents (#78835, Thermo Scientific, USA). Complete Protease Inhibitor Cocktail (#sc‐29130, Santa‐Cruz, USA), and Phosphatase Inhibitor Cocktail B (#sc‐45045, Santa‐Cruz, USA), PMSF (1 mmol/L), aprotinin (10 *μ*g/mL), leupeptin (10 *μ*g/mL), and pepstatin A (10 *μ*g/mL) were used to maintain extract integrity and function. Nuclear extracts were dialyzed using Amicon Ultra‐0.5 centrifuge filters (#UFC501096, Millipore, USA). Samples were incubated for 20 min at 4°C and centrifuged for 15 min at 20,000 g. The protein content of the supernatants was quantified using a Quick Start Bradford Protein Assay (Bio‐Rad Laboratories, USA). The samples were diluted in Laemmli buffer. Total protein sample (40 *μ*g/lane) was run on 10% SDS‐PAGE and transferred to a nitrocellulose membrane (Bio‐Rad Laboratories, USA). Membranes were blocked for 1 h at room temperature with blocking buffer (5% nonfat milk powder, TBS pH 7.4, and 0.1% Tween‐20) and incubated overnight at 4°C with the primary monoclonal antibodies. We used primary antibodies against Akt (1:1000; #2920, Cell Signaling, USA) and pAkt (Ser 473; 1:1000, #4058, Cell Signaling, USA), Bcl‐3 (1:2000, #sc‐185, Santa Cruz, USA), I*κ*B*α* (1:1000, #9242, Cell Signaling, USA), MuRF1 (1:1000, ab183094, Abcam, USA), NF‐kB p105/p50 (1:1000, #13586, Cell Signaling, USA), and pFoxO3 (Ser 253; 1:1000; #sc‐101683, Santa Cruz, USA). Blots incubated with antibodies against GAPDH (1:10,000, #G041, ABM, Canada) were used for the normalization of loading for total muscle lysates and for the cytoplasmic fractions. Nuclear fractions were normalized to the Lamin B1 content (1:1000, ab16048, Abcam, USA) in each sample. Images of nitrocellulose membranes stained with Ponceau S were used to verify equal protein loading in each lane. There were no differences in GAPDH protein content among control, HS, and HS+IMD groups when normalized for the total protein in each lane using Ponceau S‐stained membranes.

After three washes (10 min each) with TBS‐Tween (TBS and 0.1% Tween‐20), the membranes were incubated for 1 h at room temperature with horseradish peroxidase‐conjugated goat antirabbit (1:30,000, #111‐035‐003, Jackson Immuno Research, USA) or goat antimouse (1:25,000, #5178‐2504, Bio‐Rad, USA) secondary antibodies. The membranes were washed again in TBS‐Tween three times, incubated with Immun‐Star HRP substrate (Bio‐Rad Laboratories, USA), and exposed to X‐ray film (Kodak, USA) using multiple exposure times. Protein bands were quantified using densitometry scanning (GS‐800, Quantity‐One software, BioRad Laboratories, USA). The relative content of analyzed protein in each sample was determined by normalizing band intensities to the content of GAPDH in the same sample.

### RNA isolation and reverse transcription

Total RNA was extracted from 10 mg of frozen soleus muscle samples using an RNeasy Micro Kit (Qiagen, Germany). RNA samples were treated with proteinase K and DNase I. Isolated RNA in aqueous solution was frozen at −85°C for storage.

Reverse transcription was performed by incubating 1 *μ*g of RNA, random hexamers d(N)6, dNTPs, RNase inhibitor, and MMLV reverse transcriptase for 60 min at 37°C.

### For quantitative PCR analysis

One microliter of cDNA was amplified in a 25 *μ*L SYBR Green PCR reaction containing 1 × Quantitect SYBR Green Master Mix (Syntol Moscow, Russia) and 10 *μ*mol/L of each forward and reverse primer. Sequences of the primers used in this study were as follows: MuRF1 forward 5′‐GCCAATTTGGTGCTTTTTGT‐3′, MuRF1 reversed 5′‐AAATTCAGTCCTCTCCCCGT‐3′, MAFbx forward 5′‐CTACGATGTTGCAGCCAAGA‐3′, MAFbx reversed 5′‐GGCAGTCGAGAAGTCCAGTC‐3′, *β*‐actin forward 5′‐TCATGAAGTGTGACGTTGACATCC‐3′, and *β*‐actin reversed 5′‐GTAAAACGCAGCTCAGTAACAGTC‐3′. The annealing temperature was set according to the optimal annealing temperature of PCR primers. The amplification was monitored in real time using iQ5 Multicolor Real‐Time PCR Detection System (Bio‐Rad Laboratories). To confirm the amplification specificity, PCR products from each primer pair were subjected to a melting curve analysis. Relative quantification was performed based on the threshold cycle (CT value) for each PCR sample (Livak and Schmittgen 2001). Initially two housekeeping genes were evaluated for the normalization: GAPDH and *β*‐actin. Normalization to the level of expression of GAPDH and *β*‐actin showed similar results (data not shown). *β*‐actin was chosen for the normalization of all quantitative PCR analysis experiments in this study.

### Statistical analysis

It was performed using the REST 2009 v.2.0.12 and Origin Pro v.8.0 SR5 programs at the significance level set at 0.05. Comparisons of group pairs were made using nonparametric Mann–Whitney rank‐sum test. The significance of the differences between three treatment groups was evaluated using the nonparametric version of the Newman–Keuls test for multiple comparisons. Results are given as mean±standard error of the mean.

## Results

### Effect of unloading and IMD‐0534 treatment on muscle weight

The comparison of soleus muscle weight among C, HS, and HS+IMD groups showed that inhibition of IKK*β* in vivo with IMD‐0354 during 3 days of unloading had no effect on the decrease in skeletal muscle mass. Muscle weight of soleus in C group was 93 ± 4 mg, whereas weight of soleus in both HS and HS+IMD groups was significantly reduced (73 ± 2 mg and 74 ± 1 mg, respectively, *P* < 0.05). Dry weight of soleus muscle was also lower in HS (21.4 ± 0.4 mg) and HS+IMD (21.2 ± 0.4 mg) groups when compared with control (24.1 ± 1.4 mg) (*P* < 0.05).

### Effects of unloading and IMD‐0354 treatment on the level of I*κ*B*α*

**Figure 1:**
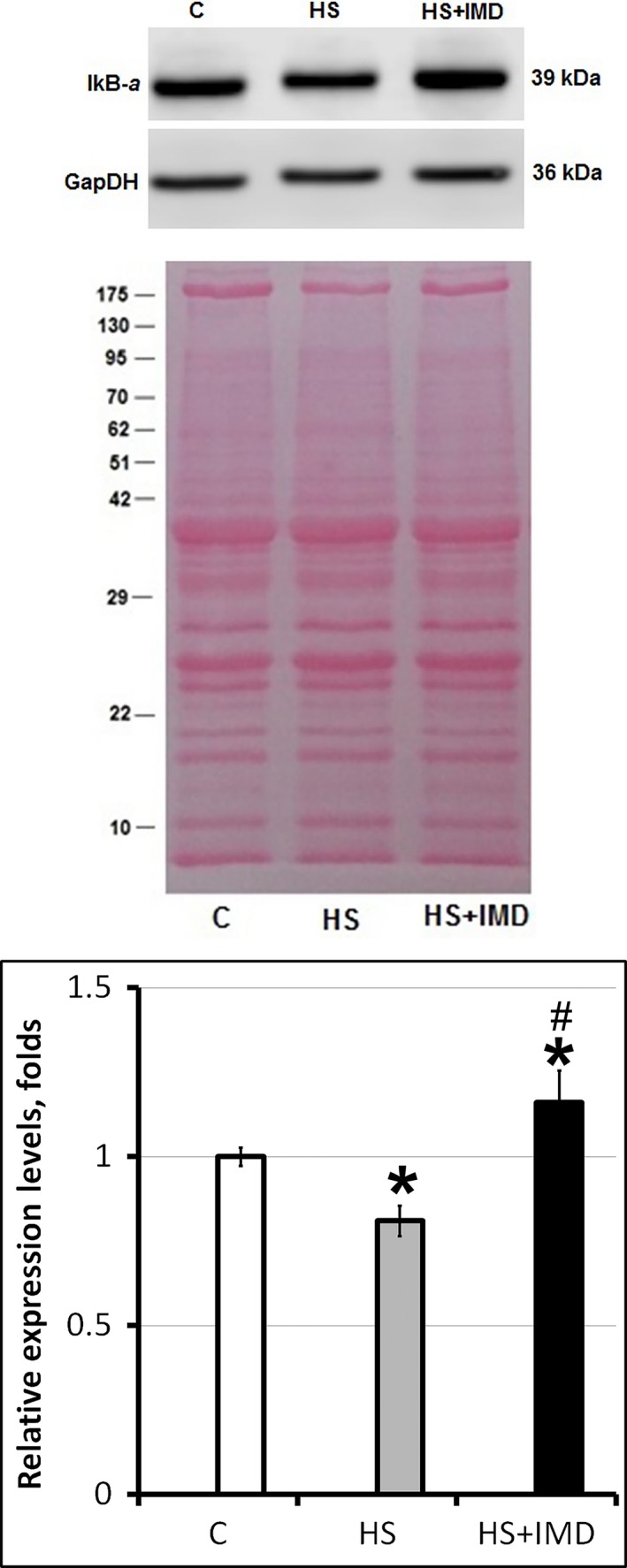
Evaluation of I*κ*B*α* protein levels in soleus muscles of C, HS, and HS+IMD rats by Western blotting. Values are normalized to the levels of total protein and GAPDH in each sample. *N* = 7. * indicates a significant difference from control, *P* < 0.05; # indicates a significant difference from HS,* P* < 0.05. Western blot image was cropped to display a single band with predicted molecular weight that was identified in our experiments. The same anti‐I*κ*B*α* antibody was used in previous publications (Ding et al. 2016; Moles et al. 2016). Images of nitrocellulose membrane stained with Ponceau S were used to verify equal protein loading in each lane.

The level of I*κ*B*α* was significantly lower in the HS group when compared with control (0.82‐fold; *P* < 0.05; Fig. 1). To the contrary, the level of I*κ*B*α* in HS+IMD group was increased when compared with the control (1.16‐fold, *P* < 0.05; Fig. 1). These data confirm that the concentration of IMD‐0354 used in our experiments was adequate for the inhibition of IKK*β* during unloading.

### Effect of unloading and IMD‐0534 treatment on the Bcl‐3, p50, and p105 transcription factors

**Figure 2:**
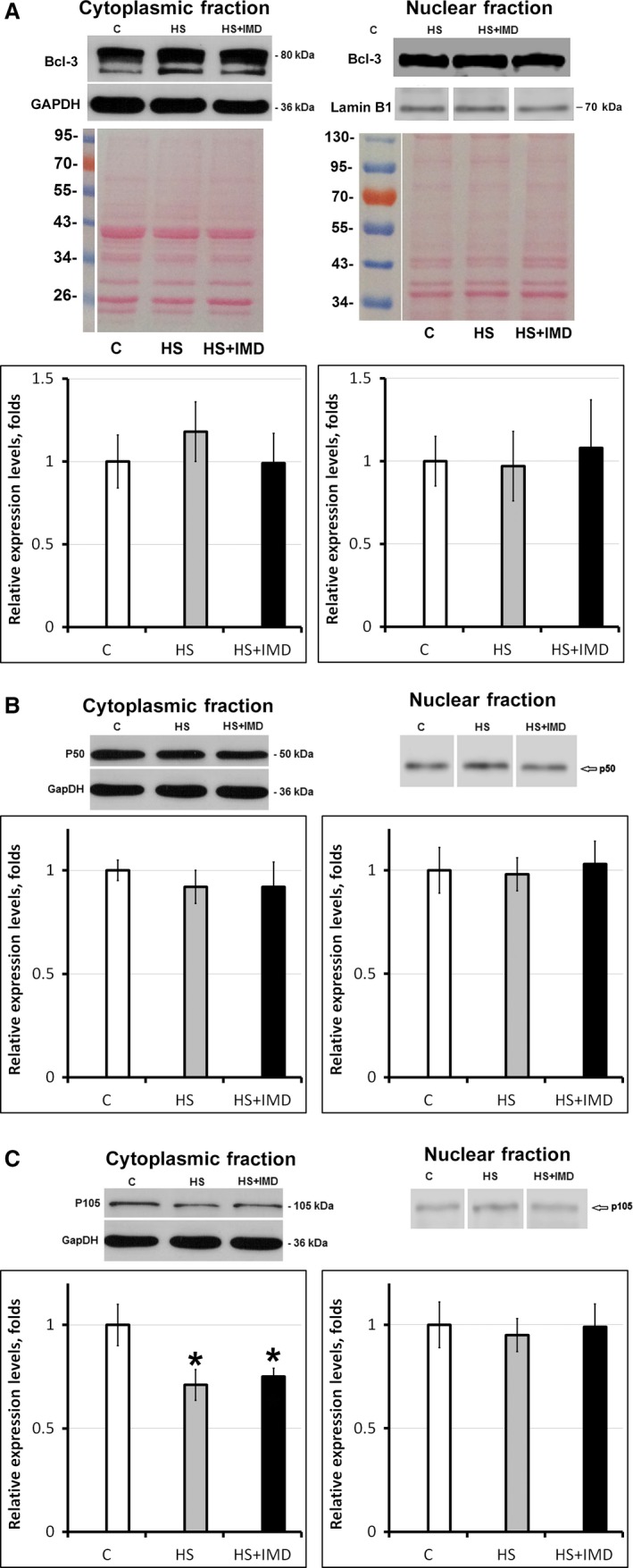
Evaluation of Bcl‐3 (A), p50 (B), and p105 (C) protein levels in soleus muscles of C, HS, and HS+IMD rats by Western blotting. Values are normalized to the levels of total protein and GAPDH (cytoplasmic fraction) or Lamin B1 (nuclear fraction) in each sample. *N* = 7. * indicates a significant difference from control, *P* < 0.05. Western blot image was cropped to display a single band with predicted molecular weight that was identified in our experiments. The same anti‐Bcl‐3 antibody (Hovelmeyer et al. 2014; Urban et al. 2016) and anti‐p50/p105 antibody (Nakazawa et al. 2016) were used in previous publications. Images of cytoplasmic and nuclear fractions of muscle protein extracts transferred to the nitrocellulose membrane and stained with Ponceau S were used to verify equal protein loading in each lane.

Bcl‐3, p50, and p105 transcription factors are involved in the NF‐*κ*B pathway‐mediated regulation of gene expression and are required for muscle atrophy. The level of Bcl‐3 was higher in the cytoplasmic fraction of skeletal muscle extracts from HS group when compared with control (1.2‐fold; Fig. 2A), although it did not reach statistically significant value. IMD‐0354 treatment prevented Bcl‐3 increase in HS+IMD‐0354 group (Fig. 2A). The levels of Bcl‐3 in the nuclear fractions of skeletal muscle extracts from HS groups with/without IMD‐0354 were not different from control (Fig. 2A).

There was a trend for the decrease in p50 protein expression in the cytoplasmic fraction of skeletal muscle extracts from HS and HS+IMD groups when compared with control, although it has not reached the statistically significant levels (Fig. 2B). The levels of p50 protein in the nuclear fractions of skeletal muscle extracts from HS groups with/without IMD‐0354 were not different from control (Fig. 2B).

The level of p105 protein was lower in the cytoplasmic fraction of skeletal muscle extracts from both the HS and HS+IMD groups when compared with control (0.71‐fold and 0.75‐fold, respectively, *P* < 0.05; Fig. 2C). The levels of p150 protein in the nuclear fractions of skeletal muscle extracts from HS groups with/without IMD‐0354 were not different from control (Fig. 2C).

### Effects of unloading and IMD‐0354 treatment on activation of protein degradation pathways

**Figure 3:**
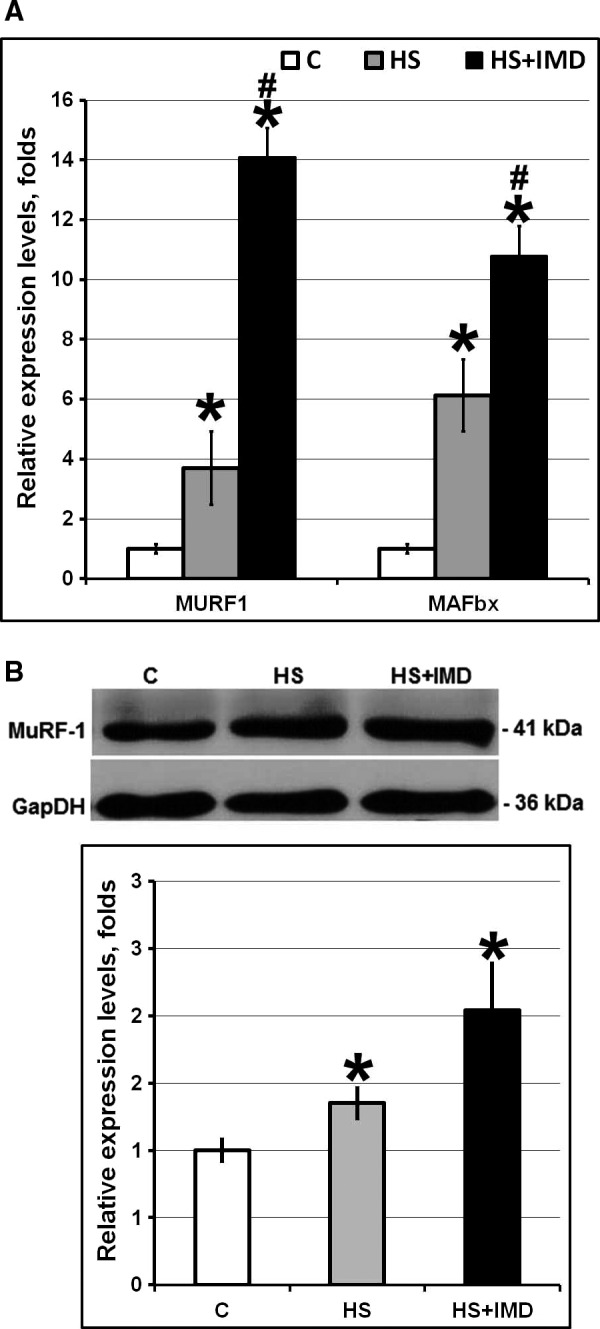
Evaluation of the levels of MuRF‐1 and MAFbx mRNA (A) and MuRF‐1 protein (B) in soleus muscles of C, HS, and HS+IMD rats. A: Evaluation of MuRF‐1 and MAFbx mRNA expression by quantitative PCR. Values are normalized to the levels of *β*‐actin mRNA in each sample. B: Evaluation of MuRF‐1 protein expression by Western blotting. Values are normalized to the levels of total protein and GAPDH in each sample. *N* = 7. * indicates a significant difference from control, *P* < 0.05; # indicates a significant difference from HS,* P* < 0.05. Western blot image was cropped to display a single band with predicted molecular weight that was identified in our experiments. Abcam web page displays a Western blot where the same anti‐MuRF‐1 antibody recognizes a single 40 kDa band.

We evaluated the levels of expression of two E3 ligases involved in protein degradation. mRNA expression of MuRF1 and MAFbx (Atrogin1) was significantly increased in both HS and HS+IMD groups when compared with control (Fig. 3A). Interestingly, the level of expression of both MuRF1 and MAFbx was significantly higher in the HS+IMD group when compared with the HS group (14.1‐fold vs. 3.7‐fold for MuRF1 and 10.1‐fold vs. 6.1‐fold for MAFbx, respectively, *P* < 0.05).

Analysis of MuRF1 protein expression levels by Western blotting showed similar results. Protein expression level of MuRF1 was significantly increased in both HS and HS+IMD groups when compared with control (Fig. 3B). We also observed a trend for the increased expression of MuRF1 protein in HS+IMD group when compared with HS group (2‐fold vs. 1.35‐fold, respectively), although it has not reached the statistically significant levels (Fig. 3B).

### Effects of unloading and IMD‐0354 treatment on the transcription factors and signaling proteins involved in muscle atrophy

**Figure 4:**
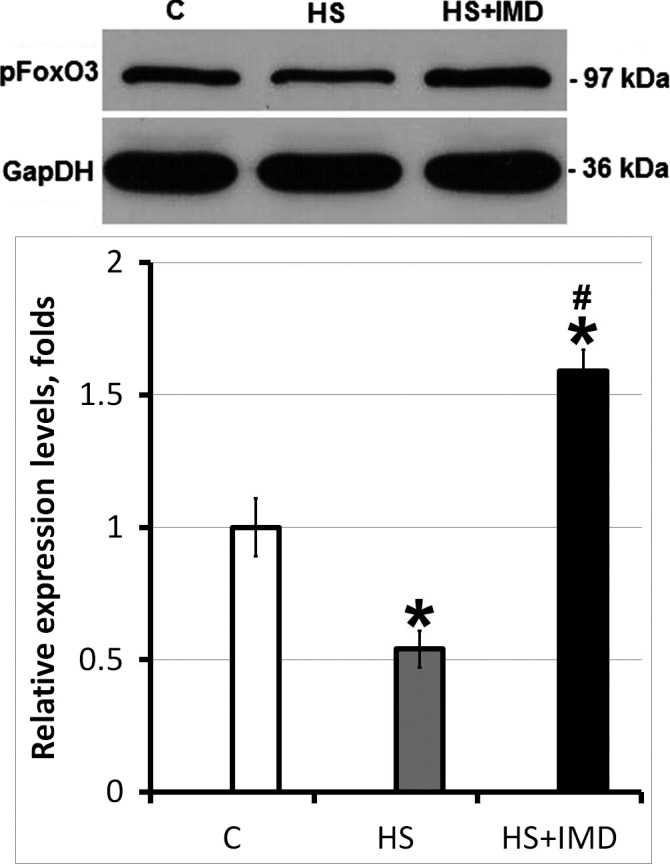
Evaluation of pFoxO3 protein level in soleus muscles of C, HS, and HS+IMD rats by Western blotting. Values are normalized to the levels of total protein and GAPDH in each sample. *N* = 7. * indicates a significant difference from control, *P* < 0.05; # indicates a significant difference from HS,* P* < 0.05. Western blot image was cropped to display a single band with predicted molecular weight that was identified in our experiments. The same anti‐pFoxO3 antibody was used in previous publications (Kukreti et al. 2013; Tarrado‐Castellarnau et al. 2015).

We evaluated the level of activation of FoxO3 transcription factor known to be involved in the regulation of MuRF1 and MAFbx expression (Fig. 4). The level of phosphorylated FoxO3*α* was significantly lower in the HS group when compared with control (0.55‐fold, *P* < 0.05). Unexpectedly, the level of pFoxO3*α* was increased in HS+IMD group when compared with control (1.6‐fold, *P* < 0.05).

**Figure 5:**
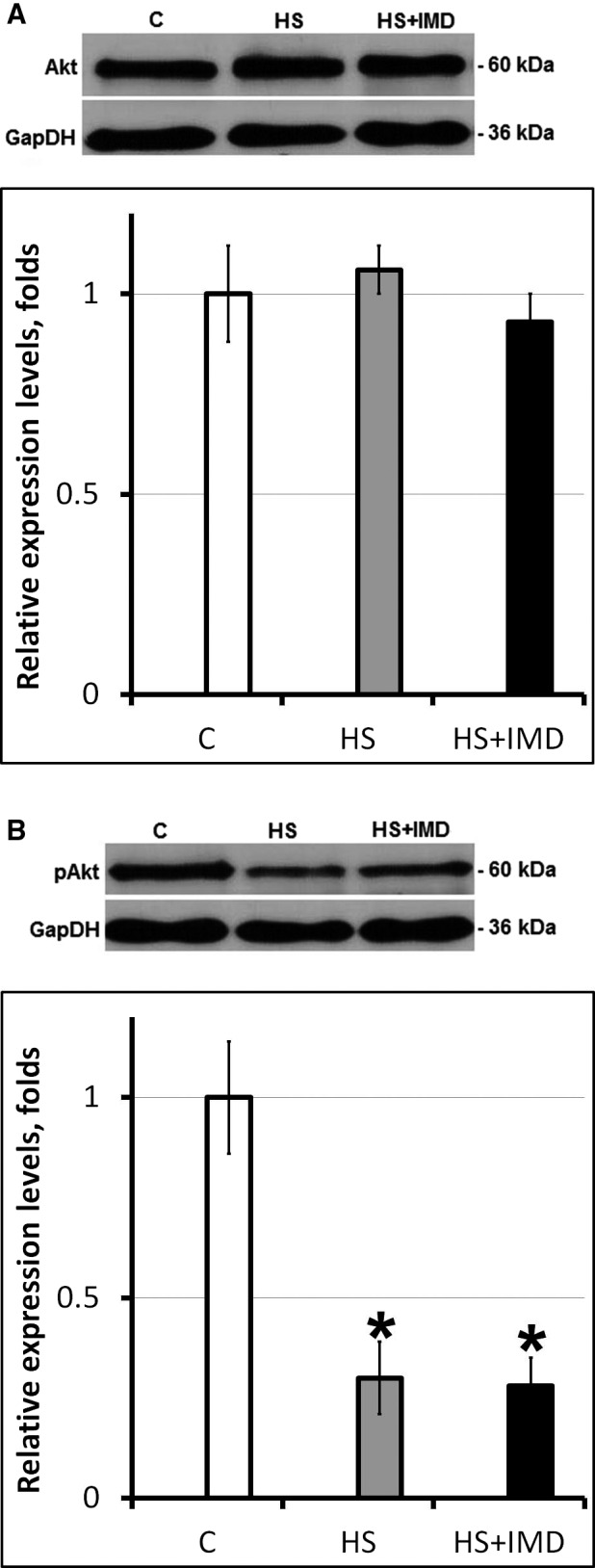
Evaluation of the total (A) and phospho‐Akt (B) protein levels in soleus muscles of C, HS, and HS+IMD rats by Western blotting. Values are normalized to the levels of total protein and GAPDH in each sample. *N* = 7. * indicates a significant difference from control, *P* < 0.05. Western blot image was cropped to display a single band with predicted molecular weight that was identified in our experiments. The same anti‐Akt (Senol‐Cosar et al. 2016; Wang et al. 2016) and phospho‐Akt (Latouche et al. 2016; Urban et al. 2016) antibodies were used in previous publications.

Activity of FoxO3*α* is negatively regulated by Akt pathway (Lokireddy et al. 2012). The levels of total Akt were not different among C, HS, and HS+IMD groups (Fig. 5A). Interestingly, despite significant difference in the levels of FoxO3*α* phosphorylation, there was no difference in pAkt content between HS and HS+IMD groups and the level of pAkt in both groups was significantly lower than in control (0.3‐fold and 0.29‐fold, respectively, *P* < 0.05; Fig. 5B).

## Discussion

### Content of I*κ*B*α* and muscle atrophy

Skeletal muscle unloading activates NF‐*κ*B pathway (Bodine and Baehr 2014; Jackman et al. 2013). However, the mechanisms of NF‐*κ*B‐mediated regulation of target genes during early stages of muscle unloading are not well studied. The majority of the previous studies were done using knockout mice or in vivo delivery of plasmids into skeletal muscle and evaluated late stages of muscle unloading. Sometimes, such approaches might interfere with the physiological processes that occur in vivo and lead to the development of the adaptive changes. For example, Xu and colleagues showed that genetic ablation of anoctamin in mice has not resulted in any overt skeletal or cardiac muscle pathology (Xu et al. 2015). In humans, mutations in this gene are associated with limb girdle muscular dystrophy and Miyoshi myopathy. Similarly, dystrophin–utrophin‐deficient mice have much milder pathology than patients with Duchenne muscular dystrophy (Moresi et al. 2010). In order to evaluate role of IKK*β* as a potential target for the development of skeletal muscle atrophy treatment it is critical to understand role of NF‐*κ*B signaling in wild‐type animals in vivo.

In our experiments, 3‐day unloading increased degradation of I*κ*B*α* . These data correlate well with previously published data on decreased I*κ*B*α* content in mice during 3‐ and 7‐day unloading (Judge et al. 2007). We showed that pharmacological inhibition of IKKβ activity using IMD‐0354 during unloading blocked I*κ*B*α* degradation. In fact, I*κ*B*α* content was higher in unloaded muscle treated with IMD‐0354 than in control muscle. Previously the same concentration of IMD‐0354 was successfully used in rats in vivo to block NF‐*κ*B pathway (Hosokawa et al. 2013). Surprisingly, preservation of the I*κ*B*α* content in unloaded muscle in our experiments has not prevented muscle atrophy. Soleus muscle mass was significantly decreased in both the unloaded group and the unloaded group treated with IMD‐0354 when compared with muscle from the control rats. This is an unexpected observation as I*κ*B*α* content in IMD‐0354‐treated unloaded muscle was significantly higher not only when compared with unloaded muscle but it was also higher than I*κ*B*α* content in the control muscle. It is known that I*κ*B*α* degradation releases NF‐*κ*B transcription factors allowing their translocation to the nuclei and resulting in atrophy (Delhase et al. 1999; Rothwarf and Karin 1999). This correlates well with our data on muscle atrophy in unloaded soleus muscle (Shenkman et al. 2015). A similar decrease in the I*κ*B*α* level of expression was reported previously after 3 and 7 days of unloading in the rat model (Judge et al. 2007). Mice with muscle‐specific transgenic expression of activated IKK*β* have profound muscle wasting due to the activation of NF‐*κ*B/MuRF (Cai et al. 2004). Muscle wasting in these mice was completely blocked by muscle‐specific expression of IkB*α* superrepressor due to the inhibition of the NF‐*κ*B pathway (Cai et al. 2004). Similarly, muscle‐specific expression of dominant‐negative I*κ*B*α* diminishes NF‐*κ*B activation, decreases levels of ubiquitinated proteins, and inhibits muscle atrophy during unloading (Judge et al. 2007). I*κ*B*α* expression vector that was used in these experiments represents N‐terminus truncated version of I*κ*B*α* that has strong inhibiting activity on the components of NF‐*κ*B pathway (Judge et al. 2007).

In contrast to the previous studies, we used a specific inhibitor of IKK*β* kinase activity during early stages of muscle unloading. IMD‐0354 prevented I*κ*B*α* degradation, but failed to inhibit unloading‐induced muscle atrophy. Interestingly, it was previously reported that despite decrease in the IkB*α* content during unloading, the level of phosphorylated I*κ*B*α* has not changed (Hunter et al. 2002; Judge et al. 2007). We hypothesize that IKK*β* and IkB*α* regulation of NF‐*κ*B activity and muscle atrophy during unloading is more complex and multifaceted than merely a simple decrease in the I*κ*B*α* content and it requires further studies. Moreover, it was reported earlier that transfection with dominant‐negative IKK*α* or IKK*β* was able to inhibit immobilization‐induced muscle atrophy by 22% while combined transfection of dominant‐negative IKK*α* and dominant‐negative FoxO3 inhibited atrophy by 89% (Reed et al. 2011). As in our experiments IMD‐0354 only prevented I*κ*B*α* degradation this probably was not sufficient for the prevention of unloading‐induced muscle atrophy in vivo.

### Transcription factors regulating MuRF1 and MAFbx expression

We tested whether inhibition of IKK*β* activity using IMD‐0354 during 3‐day unloading has an effect on FoxO3 or NF‐kB‐regulated gene expression. Unloading was characterized by the decrease in phospho‐FoxO3*α* and increase in MuRF1 (both mRNA and protein) and MAFbx (mRNA) expression. This is in agreement with our previous studies (Shenkman et al. 2015) and reports from other investigators (Bodine and Baehr 2014) demonstrating that dephosphorylation of FoxO3 promotes its translocation into the nuclei and increases MuRF1 and MAFbx expression. A decrease in phospho‐FoxO3 protein and increase in FoxO3*α* mRNA correlates with increased mRNA expression of MuRF1 and MAFbx during 3‐ and 7‐day unloading (Judge et al. 2007; Shenkman et al. 2015). It is known that Akt phosphorylates FoxO3*α* (Wang et al. 2014). Mice with muscle‐specific ablation of IKK*β* had increased phospho‐Akt level (Mourkioti et al. 2006). However, in our experiments increased phosphorylation of FoxO3 has not correlated with changes in Akt phosphorylation. Levels of phospho‐Akt were decreased in both unloaded group and unloaded group treated with IMD‐0354. At the same time, levels of phospho‐FoxO3 were significantly higher in unloaded group treated with IMD‐0354 when compared with both the unloaded group and with control group. This suggests a different source of FoxO3 phosphorylation and reflects the complexity of its regulation by IKK*β*. As the level of phospho‐FoxO3 in the unloaded group treated with IMD‐0354 was higher than in the control group, we hypothesized that under normal conditions IKK*β* kinase activity could have some inhibitory effect on FoxO3 phosphorylation. Both IKK*α* and IKK*β* are kinases and they are presumed to be able to inhibit or activate various substrates, including mTOR, FoxO3, ERK1/2, and lysosomal proteins, through phosphorylation (Jackman et al. 2013). For instance, IKK*β* phosphorylation of TSC1 activates mTOR pathway, whereas phosphorylation of FoxO3 by IKK*β* deactivates FoxO‐related transcription independently from the NF‐*κ*B pathway (Chariot 2009). Despite high level of phospho‐FoxO3 in unloaded group treated with IMD‐0354 mRNA expression of MURF‐1 and MAFbx in this group was significantly higher than in unloaded group without treatment. Similar opposing changes in the level of FoxO and the expression of E3 ubiquitin ligases were previously observed during muscle unloading (Shenkman et al. 2015). In addition to FoxO several other transcription factors can influence expression of E3 ubiquitin ligases (Bodine and Baehr 2014). For example, myogenin can activate MURF‐1 and MAFbx expression during muscle atrophy caused by denervation (Moresi et al. 2010). Moreover, recent study by Baehr et al. (2017) suggests that MURF‐1 and MAFbx are only loosely associated with protein degradation and can be upregulated during muscle unloading even in the absence of increased proteasome subunit activity.

Expression of E3 ubiquitin ligases is regulated differently during muscle unloading in studies on genetically modified IKK*α* and IKK*β* expression and in our experiments when NF‐*κ*B pathway was inhibited with IMD‐035. It was noted that inhibiting IKK*β* expression could block not only phosphorylation of I*κ*B*α* but also interfere with phosphorylation of other substrates and introduce changes in muscle physiology (Jackman et al. 2013). This could be the basis for the differences between our study and previously published data.

### Transcription factors of NF‐*κ*B pathway

Unloading‐induced muscle atrophy involves activation of NF‐*κ*B cotransactivator Bcl‐3, as well as p50, and p105 NF‐*κ*B proteins (Wu et al. 2011; Jackman et al. 2013). During muscle unloading Bcl‐3 and p50 are translocated into the nuclei, bind NF‐*κ*B‐dependent sites and activate atrophy‐related genes (Jackman et al. 2013). Both MuRF1 and MAFbx are direct targets of Bcl‐3 and p50 (Wu et al. 2011). In our experiments there were no significant changes in the Bcl‐3 content in the cytoplasm of the soleus muscle fibers following 3‐day unloading. There were also no changes in the nuclear content of Bcl‐3 in soleus muscle fibers following 3‐day unloading. It was previously demonstrated that maximal expression of both MuRF1 and MAFbx is reached after 3 days of unloading (Cannavino et al. 2014; Shenkman et al. 2015). At the same time, changes in expression of Bcl‐3 at the early stages of unloading were not reported and role of Bcl‐3 in the expression of E3 ligases during first 3 days of unloading was not clear. Previous studies demonstrated that levels of Bcl‐3 in the nuclei were significantly increased following 7 and 10 days of muscle unloading (Hunter et al. 2002; Hunter and Kandarian 2004; Judge et al. 2007) and in Bcl‐3 knockout mice atrophy of unloaded soleus muscle was significantly reduced (Hunter and Kandarian 2004). Lack of changes in cytoplasmic and nuclear content of Bcl‐3 during 3 days of unloading in our experiments suggests that during early stages of unloading other than Bcl‐3 transcription factors could be more important for muscle atrophy.

There were also no significant changes in the p50 content in the nuclei or cytoplasm of the soleus muscle fibers following 3‐day unloading in our experiments. This correlates with previous data showing no changes in p50 mRNA and protein expression during 7‐day muscle unloading (Judge et al. 2007) as well as during 2‐week muscle immobilization (Kang and JI 2013). The conservation of p50 content across the groups suggests lack of compensatory downregulation of NF‐*κ*B content in IMD‐0354‐treated rats. Therefore, in our opinion, I*κ*B*α* content represents a fair indicator of NF‐*κ*B activation.

Levels of p105 in the cytoplasmic fraction were decreased during 3 days of muscle unloading in both IMD‐0354‐treated and untreated groups in our experiments. Therefore, blocking I*κ*B*α* degradation with IMD‐0354 has no effect on the unloading‐induced decrease in p105. The reduction in p105 suggests potential downregulation of NF‐*κ*B content or accelerated NF‐*κ*B processing. The levels of p105 in the nuclear fraction were comparable between control and unloading groups with/without IMD‐0354. We have not evaluated the level of p65 in our experiments as there is no published data suggesting that the level of p65 changes at any time point after muscle unloading. It is known that p65 subunit is not required for the disuse atrophy (Jackman et al. 2013). At 6 days of skeletal muscle unloading, there was a decrease in p65 binding to *κ*B sites of both MURF‐1 and MAFbx genes in ChIP assay (Wu et al. 2011). Based on these data we suggest that p65 does not play a critical role in regulation of MURF‐1 and MAFbx expression at early stages of muscle unloading.

In summary, after 3 days of unloading we observed atrophy of soleus muscle and significant increase in the expression of MURF‐1 and MAFbx without significant changes in Bcl‐3 and p50. Interestingly, despite lack of changes in Bcl‐3 and p50 expression during unloading IMD‐0354‐treated group had higher levels of MURF‐1 and MAFbx expression than untreated group. This suggests that at early stages of muscle unloading the expression of MURF‐1 and MAFbx could be regulated by different transcription factors. This correlates well with previous data showing that skeletal muscle immobilization due to external fixation has a biphasic effect on NF‐kB activation with initial decrease in activity at early stages and subsequent increase at the later stages (Bar‐Shai et al. 2005). It was previously suggested that decreased muscle atrophy mediated by genetic inhibition of NF‐*κ*B signaling proteins can be attributed to the effects unrelated to the NF‐*κ*B transcription factors (Jackman et al. 2013).

Overall, our study showed that inhibition of IKK*β* in vivo using IMD‐0354 during 3‐day unloading prevented I*κ*B*α* degradation in soleus muscle, but failed to diminish expression of MuRF1 and MAFbx ubiquitin ligases and muscle atrophy. The review of all available publications where IMD‐0354 was used in animal models showed that this inhibitor does not have indirect or systemic effects with negative consequences for the health of the animals (Maslanka et al. 2016; Zhou et al. 2016). Therefore, we do not think that our observations are due to the systemic effects of IMD‐0354 during unloading. Further studies are needed to determine factors important for the regulation of MuRF1 and MAFbx expression during early stages of unloading.

## Conflict of Interest

All authors approve this submission. None of the authors of this manuscript have any conflicts of interest to report.

## References

[phy213291-bib-0001] Baehr, L. M. , D. W. West , A. G. Marshall , G. R. Marcotte , K. Baar , and S. C. Bodine . 2017 Muscle‐specific and age‐related changes in protein synthesis and protein degradation in response to hindlimb unloading in rats. J. Appl. Physiol. doi: 10.1152/japplphysiol.00703.2016 [Epub ahead of print]. 10.1152/japplphysiol.00703.2016 PMC5451534 28336537

[phy213291-bib-0002] Bar‐Shai, M. , E. Carmeli , R. Coleman , N. Rozen , S. Perek , D. Fuchs , et al. 2005 The effect of hindlimb immobilization on acid phosphatase, metalloproteinases and nuclear factor‐kappaB in muscles of young and old rats. Mech. Ageing Dev. 126:289–297. 15621209 10.1016/j.mad.2004.08.030

[phy213291-bib-0003] Bodine, S. C. , and L. M. Baehr . 2014 Skeletal muscle atrophy and the E3 ubiquitin ligases MuRF1 and MAFbx/atrogin‐1. Am. J. Physiol. Endocrinol. Metab. 307:E469–E484. 25096180 10.1152/ajpendo.00204.2014 PMC4166716

[phy213291-bib-0004] Cai, D. , J. D. Frantz , N. E. Tawa JR. , P. A. Melendez , B. C. Oh , H. G. Lidov , et al. 2004 IKKbeta/NF‐kappaB activation causes severe muscle wasting in mice. Cell 119:285–298. 15479644 10.1016/j.cell.2004.09.027

[phy213291-bib-0005] Cannavino, J. , L. Brocca , M. Sandri , R. Bottinelli , and M. A. Pellegrino . 2014 PGC1‐alpha over‐expression prevents metabolic alterations and soleus muscle atrophy in hindlimb unloaded mice. J. Physiol. 592:4575–4589. 25128574 10.1113/jphysiol.2014.275545 PMC4287741

[phy213291-bib-0006] Chariot, A. 2009 The NF‐kappaB‐independent functions of IKK subunits in immunity and cancer. Trends Cell Biol. 19:404–413. 19648011 10.1016/j.tcb.2009.05.006

[phy213291-bib-0007] Delhase, M. , M. Hayakawa , Y. Chen , and M. Karin . 1999 Positive and negative regulation of IkappaB kinase activity through IKKbeta subunit phosphorylation. Science 284:309–313. 10195894 10.1126/science.284.5412.309

[phy213291-bib-0008] Ding, S. , N. Mooney , B. Li , M. R. Kelly , N. Feng , A. V. Loktev , et al. 2016 Comparative proteomics reveals strain‐specific beta‐TrCP degradation via rotavirus NSP1 Hijacking a host Cullin‐3‐Rbx1 complex. PLoS Pathog. 12:e1005929. 27706223 10.1371/journal.ppat.1005929 PMC5051689

[phy213291-bib-0009] van Gammeren, D. , J. S. Damrauer , R. W. Jackman , and S. C. Kandarian . 2009 The IkappaB kinases IKKalpha and IKKbeta are necessary and sufficient for skeletal muscle atrophy. FASEB J. 23:362–370. 18827022 10.1096/fj.08-114249 PMC2630783

[phy213291-bib-0010] Hosokawa, S. , G. Haraguchi , A. Sasaki , H. Arai , S. Muto , A. Itai , et al. 2013 Pathophysiological roles of nuclear factor kappaB (NF‐kB) in pulmonary arterial hypertension: effects of synthetic selective NF‐kB inhibitor IMD‐0354. Cardiovasc. Res. 99:35–43. 23631839 10.1093/cvr/cvt105

[phy213291-bib-0011] Hovelmeyer, N. , M. A. Worns , S. Reissig , P. Adams‐Quack , J. Leclaire , M. Hahn , et al. 2014 Overexpression of Bcl‐3 inhibits the development of marginal zone B cells. Eur. J. Immunol. 44:545–552. 24242374 10.1002/eji.201343655

[phy213291-bib-0012] Hunter, R. B. , and S. C. Kandarian . 2004 Disruption of either the Nfkb1 or the Bcl3 gene inhibits skeletal muscle atrophy. J. Clin. Invest. 114:1504–1511. 15546001 10.1172/JCI21696 PMC525738

[phy213291-bib-0013] Hunter, R. B. , E. Stevenson , A. Koncarevic , H. Mitchell‐Felton , D. A. Essig , and S. C. Kandarian . 2002 Activation of an alternative NF‐kappaB pathway in skeletal muscle during disuse atrophy. FASEB J. 16:529–538. 11919155 10.1096/fj.01-0866com

[phy213291-bib-0014] Jackman, R. W. , E. W. Cornwell , C. L. Wu , and S. C. Kandarian . 2013 Nuclear factor‐kappaB signalling and transcriptional regulation in skeletal muscle atrophy. Exp. Physiol. 98:19–24. 22848079 10.1113/expphysiol.2011.063321 PMC3505235

[phy213291-bib-0015] Judge, A. R. , A. Koncarevic , R. B. Hunter , H. C. Liou , R. W. Jackman , and S. C. Kandarian . 2007 Role for IkappaBalpha, but not c‐Rel, in skeletal muscle atrophy. Am. J. Physiol. Cell Physiol. 292:C372–C382. 16928772 10.1152/ajpcell.00293.2006

[phy213291-bib-0016] Kaisari, S. , O. Rom , D. Aizenbud , and A. Z. Reznick . 2013 Involvement of NF‐kappaB and muscle specific E3 ubiquitin ligase MuRF1 in cigarette smoke‐induced catabolism in C2 myotubes. Adv. Exp. Med. Biol. 788:7–17. 23835952 10.1007/978-94-007-6627-3_2

[phy213291-bib-0017] Kang, C. , and L. L. JI . 2013 Muscle immobilization and remobilization downregulates PGC‐1alpha signaling and the mitochondrial biogenesis pathway. J. Appl. Physiol. (1985) 115:1618–1625. 23970536 10.1152/japplphysiol.01354.2012

[phy213291-bib-0018] Kukreti, H. , K. Amuthavalli , A. Harikumar , S. Sathiyamoorthy , P. Z. Feng , R. Anantharaj , et al. 2013 Muscle‐specific microRNA1 (miR1) targets heat shock protein 70 (HSP70) during dexamethasone‐mediated atrophy. J. Biol. Chem. 288:6663–6678. 23297411 10.1074/jbc.M112.390369 PMC3585106

[phy213291-bib-0019] Latouche, C. , A. Natoli , M. Reddy‐Luthmoodoo , S. E. Heywood , J. A. Armitage , and B. A. Kingwell . 2016 MicroRNA‐194 modulates glucose metabolism and its skeletal muscle expression is reduced in diabetes. PLoS ONE 11:e0155108. 27163678 10.1371/journal.pone.0155108 PMC4862646

[phy213291-bib-0020] Livak, K. J. , and T. D. Schmittgen . 2001 Analysis of relative gene expression data using real‐time quantitative PCR and the 2(‐Delta Delta C(T)) Method. Methods 25:402–408. 11846609 10.1006/meth.2001.1262

[phy213291-bib-0021] Lokireddy, S. , I. W. Wijesoma , S. K. Sze , C. McFarlane , R. Kambadur , and M. Sharma . 2012 Identification of atrogin‐1‐targeted proteins during the myostatin‐induced skeletal muscle wasting. Am. J. Physiol. Cell Physiol. 303:C512–C529. 22673621 10.1152/ajpcell.00402.2011

[phy213291-bib-0022] Lomonosova, Y. N. , B. S. Shenkman , and T. L. Nemirovskaya . 2012 Attenuation of unloading‐induced rat soleus atrophy with the heat‐shock protein inducer 17‐(allylamino)‐17‐demethoxygeldanamycin. FASEB J. 26:4295–4301. 22751006 10.1096/fj.12-204412

[phy213291-bib-0023] Maslanka, T. , I. Otrocka‐Domagala , M. Zuska‐Prot , M. Mikiewicz , J. Przybysz , A. Jasiecka , et al. 2016 IkappaB kinase beta inhibitor, IMD‐0354, prevents allergic asthma in a mouse model through inhibition of CD4(+) effector T cell responses in the lung‐draining mediastinal lymph nodes. Eur. J. Pharmacol. 775:78–85. 26868187 10.1016/j.ejphar.2016.02.023

[phy213291-bib-0024] McFarlane, C. , E. Plummer , M. Thomas , A. Hennebry , M. Ashby , N. Ling , et al. 2006 Myostatin induces cachexia by activating the ubiquitin proteolytic system through an NF‐kappaB‐independent, FoxO1‐dependent mechanism. J. Cell. Physiol. 209:501–514. 16883577 10.1002/jcp.20757

[phy213291-bib-0025] Moles, A. , J. A. Butterworth , A. Sanchez , J. E. Hunter , J. Leslie , H. Sellier , et al. 2016 A RelA(p65) Thr505 phospho‐site mutation reveals an important mechanism regulating NF‐kappaB‐dependent liver regeneration and cancer. Oncogene 35:4623–4632. 26853469 10.1038/onc.2015.526 PMC4862573

[phy213291-bib-0026] Moresi, V. , A. H. Williams , E. Meadows , J. M. Flynn , M. J. Potthoff , J. McAnally , et al. 2010 Myogenin and class II HDACs control neurogenic muscle atrophy by inducing E3 ubiquitin ligases. Cell 143:35–45. 20887891 10.1016/j.cell.2010.09.004 PMC2982779

[phy213291-bib-0027] Mourkioti, F. , P. Kratsios , T. Luedde , Y. H. Song , P. Delafontaine , R. Adami , et al. 2006 Targeted ablation of IKK2 improves skeletal muscle strength, maintains mass, and promotes regeneration. J. Clin. Invest. 116:2945–2954. 17080195 10.1172/JCI28721 PMC1626136

[phy213291-bib-0028] Nakazawa, S. , D. Oikawa , R. Ishii , T. Ayaki , H. Takahashi , H. Takeda , et al. 2016 Linear ubiquitination is involved in the pathogenesis of optineurin‐associated amyotrophic lateral sclerosis. Nat. Commun. 7:12547. 27552911 10.1038/ncomms12547 PMC4999505

[phy213291-bib-0029] Reed, S. A. , S. M. Senf , E. W. Cornwell , S. C. Kandarian , and A. R. Judge . 2011 Inhibition of IkappaB kinase alpha (IKKalpha) or IKKbeta (IKKbeta) plus forkhead box O (Foxo) abolishes skeletal muscle atrophy. Biochem. Biophys. Res. Commun. 405:491–496. 21256828 10.1016/j.bbrc.2011.01.059 PMC3056397

[phy213291-bib-0030] Rothwarf, D. M. , and M. Karin . 1999 The NF‐kappa B activation pathway: a paradigm in information transfer from membrane to nucleus. Sci STKE 1999:RE1. 11865184 10.1126/stke.1999.5.re1

[phy213291-bib-0031] Senol‐Cosar, O. , R. J. Flach , M. Distefano , A. Chawla , S. Nicoloro , J. Straubhaar , et al. 2016 Tenomodulin promotes human adipocyte differentiation and beneficial visceral adipose tissue expansion. Nat. Commun. 7:10686. 26880110 10.1038/ncomms10686 PMC4757769

[phy213291-bib-0032] Shenkman, B. S. , S. P. Belova , Y. N. Lomonosova , T. Y. Kostrominova , and T. L. Nemirovskaya . 2015 Calpain‐dependent regulation of the skeletal muscle atrophy following unloading. Arch. Biochem. Biophys. 584:36–41. 26297661 10.1016/j.abb.2015.07.011

[phy213291-bib-0033] Smith, H. K. , K. G. Matthews , J. M. Oldham , F. Jeanplong , S. J. Falconer , J. J. Bass , et al. 2014 Translational signalling, atrogenic and myogenic gene expression during unloading and reloading of skeletal muscle in myostatin‐deficient mice. PLoS ONE 9:e94356. 24718581 10.1371/journal.pone.0094356 PMC3981781

[phy213291-bib-0034] Tarrado‐Castellarnau, M. , R. Cortes , M. Zanuy , J. Tarrago‐Celada , I. H. Polat , R. Hill , et al. 2015 Methylseleninic acid promotes antitumour effects via nuclear FOXO3a translocation through Akt inhibition. Pharmacol. Res. 102:218–234. 26375988 10.1016/j.phrs.2015.09.009 PMC4850087

[phy213291-bib-0035] Urban, B. C. , T. J. Collard , C. J. Eagle , S. L. Southern , A. Greenhough , M. Hamdollah‐Zadeh , et al. 2016 BCL‐3 expression promotes colorectal tumorigenesis through activation of AKT signalling. Gut 65:1151–1164. 26033966 10.1136/gutjnl-2014-308270 PMC4941180

[phy213291-bib-0036] Wang, Y. , Y. Zhou , and D. T. Graves . 2014 FOXO transcription factors: their clinical significance and regulation. Biomed. Res. Int. 2014:925350. 24864265 10.1155/2014/925350 PMC4016844

[phy213291-bib-0037] Wang, Y. , Y. Li , L. Song , Y. Li , S. Jiang , and S. Zhang . 2016 The transplantation of Akt‐overexpressing amniotic fluid‐derived mesenchymal stem cells protects the heart against ischemia‐reperfusion injury in rabbits. Mol. Med. Rep. 14:234–242. 27151366 10.3892/mmr.2016.5212 PMC4918560

[phy213291-bib-0038] Wu, C. L. , S. C. Kandarian , and R. W. Jackman . 2011 Identification of genes that elicit disuse muscle atrophy via the transcription factors p50 and Bcl‐3. PLoS ONE 6:e16171. 21249144 10.1371/journal.pone.0016171 PMC3020958

[phy213291-bib-0039] Wu, C. L. , E. W. Cornwell , R. W. Jackman , and S. C. Kandarian . 2014 NF‐kappaB but not FoxO sites in the MuRF1 promoter are required for transcriptional activation in disuse muscle atrophy. Am. J. Physiol. Cell Physiol. 306:C762–C767. 24553183 10.1152/ajpcell.00361.2013 PMC3989716

[phy213291-bib-0040] Xu, J. , M. el Refaey , L. Xu , L. Zhao , Y. Gao , K. Floyd , et al. 2015 Genetic disruption of Ano5 in mice does not recapitulate human ANO5‐deficient muscular dystrophy. Skelet Muscle 5:43. 26693275 10.1186/s13395-015-0069-z PMC4685631

[phy213291-bib-0041] Zhou, Y. , Y. Hong , and H. Huang . 2016 Triptolide attenuates inflammatory response in membranous glomerulo‐nephritis rat via downregulation of nf‐kappab signaling pathway. Kidney Blood Press. Res. 41:901–910. 27871079 10.1159/000452591

